# Academy of Medicine—Section on Theory and Practice

**Published:** 1879-07

**Authors:** 


### ACADEMY OF MEDICINE—SECTION ON THEORY AND PRACTICE.

NEW YORK, May 20, 1879.

At 9 P.M. the Section was called to order by Dr. E. H. M. Sell, chairman.

It was voted that, owing to the lateness of the hour, the reading of the minutes of the last meeting be omitted.

The chairman announced as the subject for the evening, the continuation of the subject of the last meeting, viz.: "Changes in Type and Treatment of Disease in the last thirty years," and called upon Dr. J. C. Peters to finish the paper, of which he had read a portion at the last meeting of the Section.

Dr. Peters said that among the many causes to which have been attributed a change in the type of disease were the great famine in Europe, which introduced the typhoid element, the great epidemics of cholera, the weather, and the treatment of disease. Dr. Peters read a carefully prepared paper, giving many statistics, and illustrating the effect which each of these causes had had upon the type of disease; and it is his belief that there had been no permanent change in the type of disease, with the possible exception of scarlet fever. Under the same conditions and treatment, the type of disease remains the same. Treatment of disease had, however, during the last thirty years, changed very much, and the change was due to an advance in knowledge. The average practitioner is a better diagnostician to day than he was thirty years ago.

Blood-letting and the excessive use of mercury have been discontinued in diseases of an asthenic type. Opium treatment in inflammatory diseases has been introduced. Dr. Peters, many years ago, called the attention of the profession to the fact that drunkards seldom died of phthisis, but that, in the great number of autopsies he had made of this class, many old cicatrices were found in the lungs. Since that time alcohol has been successfully used in the treatment of phthisis. Some of the old, discarded remedies are being revived—notably, blood-letting and the use of calomel in large, so-called sedative doses. Homœpathy had the effect of developing the expectant plan of treatment.

Dr. J. Lewis Smith agrees with Dr. Peters that, on the whole, medical men are better diagnosticians than they were formerly, and that they have a more thorough knowledge of pathology, and give more careful and accurate descriptions of disease. This has led to changes in names. True, croup he does not believe to be more prevalent now than formerly, but thinks some of that which is called croup at present, is diphtheria; and thinks the two diseases to be entirely distinct. Within his experience the type of disease has not changed.

Dr. Messinger stated that in his time he had not seen much change in the type of disease.

Dr. H. P. Farnum, referring to the revival of old remedies, said he had found that the sedative dose (ʒss ) of calomel was not followed by catharsis, and asked what the experience of members of the Section had been in that particular. The remarks of several members, in reply to Dr. Farnum's question, indicated that the exhibition of thirty or forty grains of calomel is usually followed by free catharsis.

Dr. A. S. Church remarked that the theory of change of type in disease from the influence of epidemics he believed to be untenable, but would attribute more of the apparent change to the different modes of living and surrounding circumstances, and to the different methods of treatment adopted. In addition to the causes which had influenced treatment, already mentioned by Dr. Peters, he thought public opinion might be mentioned, and especially in regard to blood-letting, which, he thought, ought sometimes to be resorted to when it was not. He did not believe that there had been any change in the type of disease, but that under the same circumstances it would always present the same symptoms and require the same treatment.

Dr. Kennedy thought blood-letting was abandoned, because of homœopathy, and ought to be revived. He did not believe there had been a change in the type of disease. Cholera is, and always has been, the same.

The Section then adjourned until the call of the chairman, when Prof. J. Lewis Smith, M.D., has consented to read a paper, the subject to be announced in due time.—*The Physician and Pharmacist.*

